# Determining Accurate Pore Structures of Polypropylene Membrane for ECMO Using FE-SEM Under Optimized Conditions

**DOI:** 10.3390/membranes15060174

**Published:** 2025-06-09

**Authors:** Makoto Fukuda, Yoshiaki Nishite, Eri Murata, Koki Namekawa, Tomohiro Mori, Tsutomu Tanaka, Kiyotaka Sakai

**Affiliations:** 1 Department of Biomedical Engineering, Kindai University, 930 Nishimitani, Kinokawa-City 649-6493, Wakayama, Japan; menisite@waka.kindai.ac.jp; 2 Graduate School of Biology-Oriented Science and Technology, Kindai University, 930 Nishimitani, Kinokawa-City 649-6493, Wakayama, Japan; 3 Department of Clinical Engineering, Juntendo University, 6-8-1 Hinode, Urayasu-City 279-0013, Chiba, Japan; k.namekawa.at@juntendo.ac.jp; 4 Industrial Technology Center of Wakayama Prefecture, 60 Ogura, Wakayama-City 649-6261, Wakayama, Japan; tomohiro_mori@wakayama-kg.jp; 5 Osaka Research Institute of Industrial Science and Technology, 2-7-1 Ayumino, Izumi-City 594-1157, Osaka, Japan; t_tanaka@orist.jp; 6 School of Advanced Science and Engineering, Waseda University, 3-4-1 Okubo, Shinjuku-ku 169-8555, Tokyo, Japan; kisakai@waseda.jp

**Keywords:** extracorporeal membrane oxygenator, pore structure, polypropylene, field emission scanning electron microscopy, sputter coating

## Abstract

Long-term ECMOs are expected to be put into practical use in order to prepare for the next emerging severe infectious diseases after the novel coronavirus pandemic in 2019–2023. While polypropylene (PP) and polymethylpentene (PMP) are currently the mainstream materials for the hollow fiber membranes of ECMO, the PP membrane coated with a silicone layer on the outer surface has also been commercialized. In this study, we sought a method to accurately observe the detailed pore morphologies of the PP membrane by suppressing irreversible changes in the morphology in SEM observation, which is a general-purpose observation with higher resolution. As a result, the convex surface morphologies of the PP membrane, which was a non-conductive porous structure, were confirmed in detail by utilizing the lower secondary electron image (LEI) mode (FE-SEM, JSM-7610F, JEOL Ltd., Tokyo, Japan) at low acceleration voltage, low magnification, and long working distance, to minimize morphological alterations caused by osmium (Os) sputtering. On the other hand, although the sputter-coating on non-conductive samples is mandatory for imaging morphologies with SEM, the non-sputtering method is also worthwhile for porous and fragile structures such as this sample to minimize morphological alterations. Furthermore, we propose a method to confirm the morphology of the deep part of the sample by utilizing the secondary electron image (SEI) mode at an appropriate acceleration voltage and high magnification with higher resolution.

## 1. Introduction

The extracorporeal membrane oxygenator (ECMO) is an artificial organ that has attracted worldwide attention after the novel coronavirus pandemic in 2019–2023 [1]. The requirements for the membrane have both high gas permeability and low water vapor permeability, while the development of ECMO for long-term usage is anticipated [2,3,4,5].

There was concern that SARS-CoV-2 in plasma penetrated through the pore in the hollow fiber membrane and diffused as an aerosol from the gas outlet port of ECMO during the treatment of COVID-19 severely ill patients with ECMO [6]. We previously used atomic force microscopy (AFM) to clarify the pore structure of ECMO membranes by using our approach and theoretically validate the risk of SARS-CoV-2 permeation [7]. However, the surface roughness of the membranes was larger than the amplitude of the probes, and we did not obtain appropriate data for polypropylene (PP) membranes for ECMO [7]. Therefore, field emission scanning electron microscopy (FE-SEM) has been used to clarify the pore structures of the membranes, and theoretically determine the permeability of SARS-CoV-2 through the pores [7]. Furthermore, the pore structures of the walls of the cross-sectional membranes made from polymethypentene (PMP) were clarified utilizing the ion-milling (IM) method [8,9], which did not apply physical stress to the porous structure when cutting the wall part of the membranes [10]. The phenomena of blockage of voids in the porous structures were confirmed while using the FE-SEM, and by elevated temperature during sputter-coating of platinum (Pt) or gold (Au). Notably, the observed images of membrane morphology made from polypropylene (PP) with a lower melting point showed differences depending on the acceleration voltage and other conditions [7].

Along with the evolution of microscopic technologies such as FE-SEM [10,11], research and development for membrane science can progress by taking advantage of those techniques. The advantages and disadvantages of microscopy and spectroscopy techniques for polymeric membranes were summarized in detail [12]. In particular, the preparation of cross-sectional membrane samples has been comprehensively studied [13]. These methods have led to the evolution of practical studies of membrane porous structure–property relationship, especially in artificial organs [4].

After the novel coronavirus pandemic, studies on the structure–function relationship or antithrombotic properties of ECMO membranes have been published worldwide [14,15,16,17,18]. In addition to the typical materials such as PP and poly(4-methyl-1-pentene) [16,17,18], the composite membrane of PP and poly(4-methyl-1-pentene) [17] or the PP membrane with gradient pore structure [18] have been developed. A novel membrane based on the hollow fiber membrane made from PP coated with a silicone layer on the outer surface [5,19] has also been developed.

This study focuses on the methods to observe the pore structure of the PP membrane more precisely and in detail by utilizing an FE-SEM with different observation modes and sputter-coating methods. We explore the methods of conductive materials sputter-coating and the conditions for observation by FE-SEM, and verify the differences in the images of pore morphologies. We chose the sputtering of osmium (Os) compared to the sputtering of Au because the sputtering of Os was considered less damaging to the sample. The quantitative differences are examined in the pore diameters and distributions, and surface porosities.

## 2. Experimental Section

### 2.1. Materials

MERA NHP (SENKO MEDICAL INSTRUMENT Mfg. Co., Ltd., Tokyo, Japan) is approved as an extracorporeal membrane lung and assisted circulation/assisted respiration membrane lung for assisting respiration. The outer surface of the porous hollow fiber membrane made from PP is coated with thin silicone layer (thickness: 0.2 µm) [19], which is the extremely rare silicone-composite membrane for ECMO in the world. Inner diameter of the lumen is 246 ± 3 µm (*n* = 30), membrane thickness is 27 ± 1 µm (*n* = 30), sterilization method is EOG (ethylene oxide gas). Heparin compound is coated on the outer surface of the hollow fiber membrane for blood flow channel as an antithrombogenic material.

### 2.2. Observation of the Inner and Outer Surfaces of the Hollow Fiber Membrane Utilizing Field Emission Scanning Electron Microscopy

An FE-SEM (JSM-7610F, JEOL Ltd., Tokyo, Japan) was utilized to observe the inner and outer surfaces of the hollow fiber membrane at an accelerating voltage of 1.0, 2.0 kV, and 5.0 kV, a working distance of 15.0 or 6.0 mm, and an illumination current of 37.5 pA in observation fields of a magnification of 30,000, 50,000, and 100,000.

SEI (secondary electron image) and LEI (lower secondary electron image) modes were utilized. They differ in the position of the detector and the type of secondary electrons that are detected, and the characteristics of the images are different. In the LEI mode, since the detector is located at the same height as the sample, the LEI mode detects electrons emitted from the sample at a low angle. The depth of the field to be imaged is increased by the longer working distance (WD) making it suitable for imaging a three-dimensional structure [10]. On the other hand, in the SEI mode, since the detector is positioned above the sample, it detects low-energy secondary electrons with a shorter WD. The information on deeper parts of the sample can be obtained at high resolution by irradiating the sample with acceleration voltages from 1.0 to 30 kV. Higher acceleration voltages make it easier to obtain high-resolution images, but irreversible destruction or charge-up of the sample must be avoided.

### 2.3. Sputter-Coating of Os or Au Particles on the Inner and Outer Surfaces of the Hollow Fiber Membrane

Nonsputter-coating samples and samples with osmium (Os) or Au sputter-coating on the membranes were observed accurately and in high-resolution imaging. Various combinations of sputter-coating and observation modes were used to prevent irreversible changes in original morphologies of the samples.

Tenant 20 (MEIWAFOSIS Co., Ltd., Tokyo, Japan) was used for Os sputter-coating and Os thickness was set at 5 nm (5 mA). JFC-1500 (JEOL Ltd., Tokyo, Japan) was used for Au sputter-coating and Au thickness was set at 7 nm.

### 2.4. Determination of the Pore Diameter and Surface Porosity of the Hollow Fiber Membrane

Pore areas were measured in observation fields of magnification of 30,000 or 50,000 in size by the analysis of digital imagery utilizing Image J software (Ver.1.53, National Institute of Health, Bethesda, MD, USA). Equivalent pore diameters and surface porosities of the images were calculated using the values from pore areas. Data on the samples were treated using a paired *t*-test (*p* < 0.05).

## 3. Results and Discussion

### 3.1. FE-SEM Images of the Inner Surfaces of the PP Membrane with the Different Sputter-Coating Methods and Observation Modes

**Figure 1:**
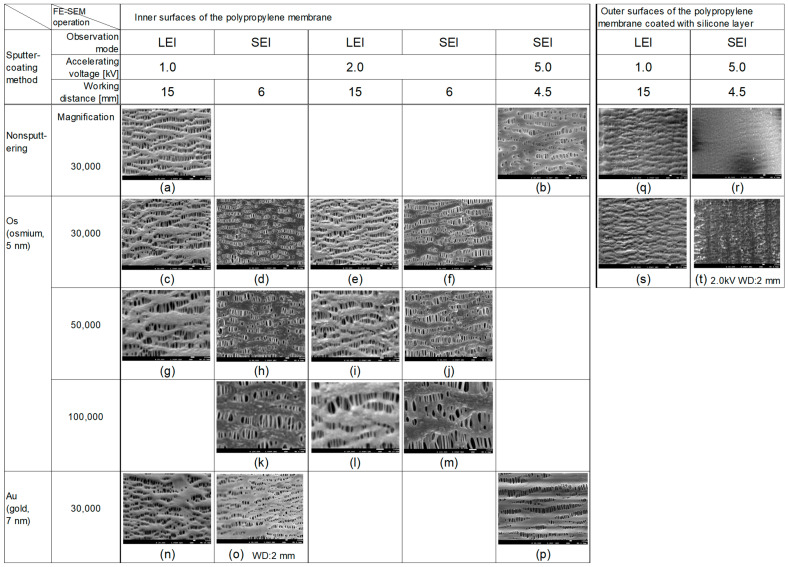
Outline of the morphologies of the inner and outer surfaces of the capillary membrane made of polypropylene coated with silicone layer on the outer surface using an FE-SEM with the different observation modes and sputter-coating methods. For example, (c) is the image of an inner surface of sputter-coating with Os in LEI mode, accelerating voltage of 1.0 kV, working distance of 15.0 mm, and magnification of 30,000.

Figure 1 shows the overview of FE-SEM images of the morphologies of the inner and outer surfaces of the PP capillary membranes under different observation modes and sputter-coating methods. The morphologies of the Os or Au sputter-coated samples were also compared to those without any sputter-coating. For example, (c) is the image of an inner surface of sputter-coating with Os in LEI mode, accelerating voltage of 1.0 kV, working distance of 15.0 mm, and magnification of 30,000. Three samples were at least prepared and observed for each condition.

**Figure 2:**
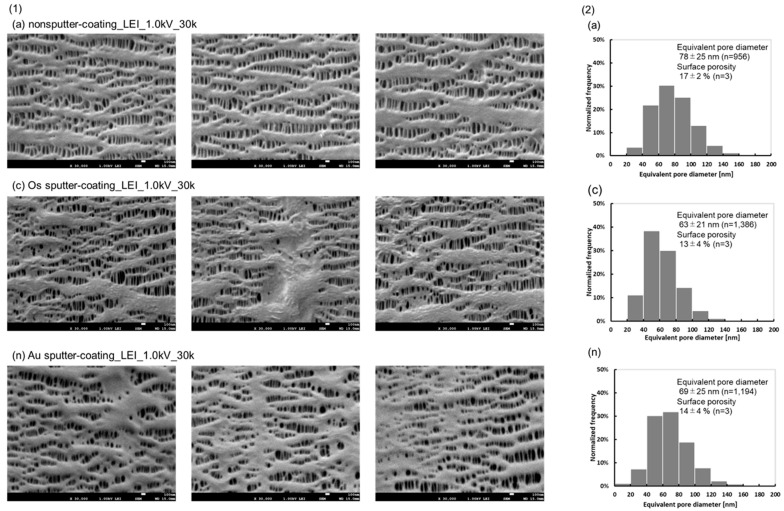
(**1**) FE-SEM images of the samples (a), (c), (n), inner surfaces of the capillary membrane, magnification; 30,000. (**2**) Distribution of the equivalent pore diameter of the samples, inner lumen of the capillary membrane determined via FE-SEM, (a), (c), and (n).

Figure 2(1) shows three images each of (a), (c) and (n). (a) is a nonsputter-coated sample, (n) is a sputter-coating with Au particles, and the other observation conditions are the same as in (c). Elongated pore structures along the longitudinal hollow fiber membrane and convex morphologies of the inner surfaces are confirmed in all images, which are characteristic of PP membranes manufactured by the stretching mechanism. These images are similar to those of the cross-sectional longitudinal structure of the PP membrane [17]. While observing the nonsputter-coated sample (a), reducing the number of secondary electrons that were detected, made it difficult to obtain the images stably. The figures of the Os sputter-coated sample (c) were stably imaged, but some of the pores were found to be occluded, and large areas composed of fibrils were specifically confirmed due to morphological alterations caused by metal sputtering. On the other hand, the Au sputter-coated images (n) showed an overall loss of convex morphology and elongated pores, and areas of the PP fibrils were large. Because of the higher conductivity of Au than Os, the morphological changes during sputtering were larger.

The differences in the images (a) (c) (n) were determined to be sputtered differences rather than differences in the micro-fiber lengths of different sections of the melt-spun stretched membranes. In terms of quality uniformity, at this frequency of sampling numbers, manufacturing of melt-spun stretched membranes (PP) does not permit the morphology in (c) and (n).

These images are different from those of the PP membrane surfaces [18]. The PP pieces were quenched in liquid nitrogen and coated with Pt using an ion sputter coater (JEC-3000FC, JEOL Ltd., Tokyo, Japan), SEM imaging at an accelerating voltage of 3 kV was used [18].

Figure 2(2) shows the distributions of the equivalent pore diameters in (a), (c) and (n). The distribution of (a) shifted slightly larger than that of (c) and (n). Significant differences in the equivalent pore diameters were found between (c) and (a) (*p* = 0.008) and between (a) and (n) (*p* = 0.021). Significant differences in the surface porosities were found between (c) and (a) (*p* = 0.013) and between (a) and (n) (*p* = 0.045).

Although charge-up occurred and the images were difficult to stabilize while observing the nonsputter-coated sample (Figure 2(1) (a)), the equivalent pore diameter and surface porosity of the observed images were significantly higher than those of (c) and (n). The slightly smaller equivalent pore diameter and surface porosity of (c) compared to those of (a) are due to the blockages of the Os sputter-coating, which reduced the size of individual pores and increased the area of the fibril. In the comparison of samples (a) and (c), the difference in the graphs shows that the average pore diameter is reduced by 15 nm on average due to the Os (5 nm) coating, which suggests that larger diameter pores are more likely to be occluded. Therefore, this can be interpreted as partial occlusion due to the Os coating.

These results demonstrate that the images of (a) in the LEI mode and at an accelerating voltage of 1.0 kV better reflect the morphology and size of the original individual pores. The relationship between different sputter-coating methods on the porous membrane made from PP and the observed images is clarified, as well as the differences in the distribution of equivalent pore diameter and surface porosity. Although the sputter-coating on the non-conductive samples is mandatory for imaging the morphologies by SEM, the non-sputtering method is worthwhile for the porous and fragile structure to minimize morphological alterations caused by metal sputtering.

**Figure 3:**
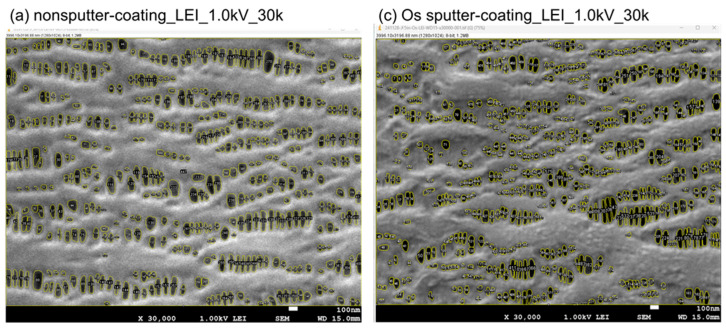
(a) Image analysis of the samples (a). (c) Image analysis of the samples (c).

Figure 3 shows the example of actual analyzed images of Figure 2(a), (c). The openings on the innermost surface of the membrane were visually checked and numbered one by one to determine if they were pores or not. Pores were differentiated from convex morphologies based on the difference in depth. The area and the circle equivalent diameter were calculated by trimming off the areas that could be recognized as pores one by one. Contrasts are slightly adjusted. The inner and outer surfaces were observed randomly, so there are no cross-sectional edges.

### 3.2. Comparison of LEI and SEI Modes

**Figure 4:**
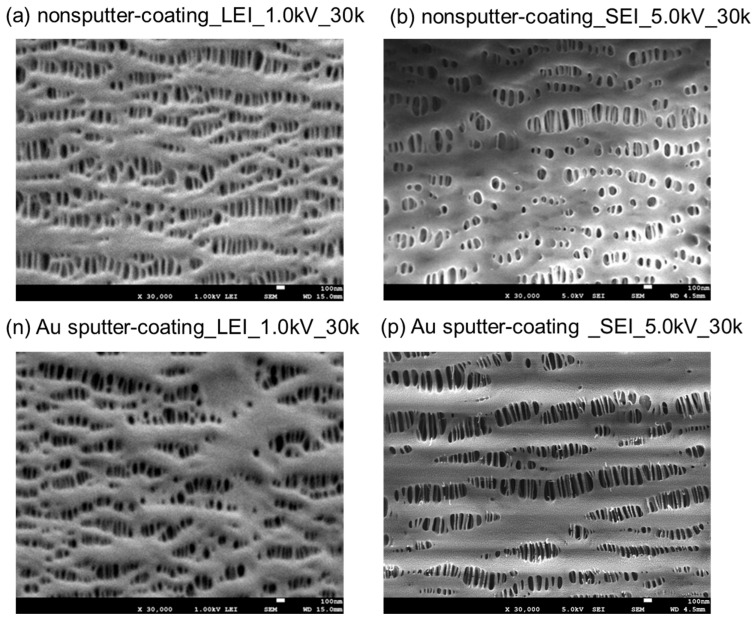
FE-SEM images of the samples ((a), (b), (n), and (p)) and inner surfaces of the capillary membrane, observed in different sputter-coating methods, modes, and acceleration voltages, magnification; 30,000.

Figure 4 shows the images in SEI mode (b)(p), compared to the nonsputter-coated sample imaged in LEI mode (a) and the Au sputter-coated sample imaged in LEI mode (n).

The morphologies of (a) and (b) were quite different. The (b) was affected by the accelerating voltage of 5.0 kV, and almost all of the elongated pores were deformed during the observation by FE-SEM, and the convex surfaces of the PP fibril chains also disappeared.

The mean values of the major axis and minor axis of (b) were 166 ± 51 nm (*n* = 50), and 53 ± 15 nm (*n* = 50), respectively [8], so the remaining pores were larger and had a larger equivalent pore diameter due to the denaturation of the pore structure, compared to the equivalent diameter of (a) (78 ± 25 nm) (*n* = 956). The Au sputter-coating image (p) also showed a characteristic morphology, differing from (n) or (a). Notably, thin PP chains were severed, pores shrunk or disappeared, and the convexity of the PP fibril chains also disappeared during FE-SEM observation. A large difference of (p) from the image in (b) was confirmed because sample (p) was only sputtered with Au with high conductivity. The results in Figure 4 indicate undesirable physical effects on the membrane, but they are remarkable outcomes.

### 3.3. Appropriate Accelerating Voltages in LEI and SEI Modes (Os Sputter-Coating)

Figure 1 compares images observed in LEI mode (c)(e)(g)(i) with those in SEI mode (d)(f)(h)(j) at accelerating voltages of 1.0 kV or 2.0 kV, for Os sputter-coating samples. From the results of Figure 1 and Figure 4, an upper limit of 2.0 kV was set as the accelerating voltage at which irreversible changes in the sample did not occur while observing the sample in SEI mode.

The images of (c) and (d) are completely different, and (d) is quite different from the typical image of the PP membrane ((a), nonsputter-coated sample). The deep morphology of the sample was not confirmed at magnification; 30,000 (d).

The images obtained by changing the accelerating voltage from 1.0 kV to 2.0 kV in each of (c) and (d) are shown in (e) and (f). The images of (e) and (f) have higher resolution than those of (c) and (d). In addition, the deep morphologies of the samples are well-defined in (f) (SEI mode) than in (e) (LEI mode). (c), (d), (e), and (f) are the ones in which the magnification factor of 30,000X, and those for (g), (h), (i) and (j) are changed to magnification; 50,000. The images of (g) and (i) (magnification; 50,000) had the same level of detail compared to (c) and (e) (LEI mode, magnification; 30,000), but (g) and (i) were slightly more indistinct.

On the other hand, (h) and (j) (SEI mode, magnification; 50,000) showed the same high resolution and detailed structure compared to (d) and (f) (SEI mode, 30,000X). In addition, the accelerating voltage of (j) was higher than that of (h), so the accurate pore morphology in the deep part of (j) was confirmed.

**Figure 5:**
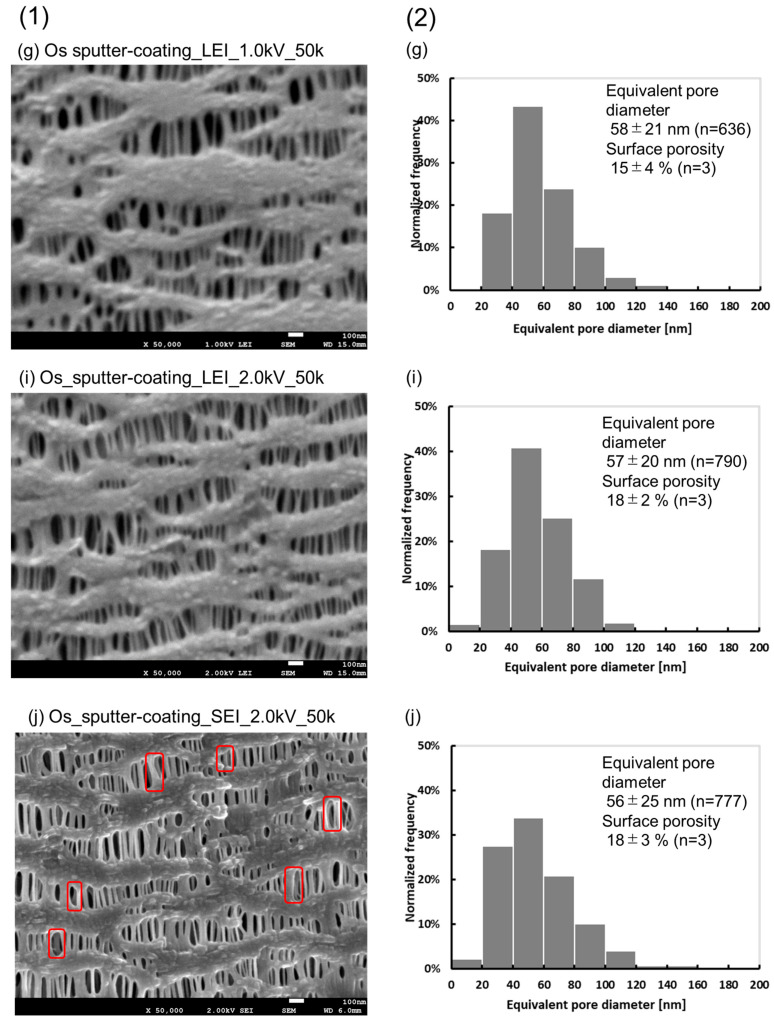
(**1**) FE-SEM images of the sample ((g), (i), and (j)), inner surfaces of the capillary membrane, observed in LEI or SEI mode, the acceleration voltages of 1.0 kV or 2.0 kV, and magnification; 50,000. (**2**) Distribution of the equivalent pore diameter of the samples, inner lumen of the capillary membrane determined via FE-SEM, (g), (i), and (j). red box: another fibril is recognized deeper than the top surface of the image.

Figure 5(1) shows a comparison of the enlarged figures of (g), (i), and (j), and Figure 5(2) shows a comparison of the distributions of their equivalent pore diameters. No significant differences between (g) and (i), and between (i) and (j) were found in either case.

The differences were obvious when comparing (i) with (j) (accelerating voltage 2.0 kV, magnification; 50,000) observed in LEI and SEI mode at a more magnified view. The image of (i) was a little blurred, while (j) had a higher resolution and more detailed structure, and the deep morphology was also confirmed. Although it is difficult to measure the depth, since another fibril is recognized deeper than the top surface of the image (red box), its depth is considered to be deeper than the thickness of the fibril of the top surface (e.g., 50 nm, 100 nm, etc.). The mean value of the surface equivalent pore diameter of (j) was 56 ± 25 nm (*n* = 777) and the surface porosity was 18 ± 3% (*n* = 3), which were not significantly different from those of (i), indicating that the surface morphology was equivalent. These results demonstrate that the deep morphology and precise structure are clearly confirmed with high resolution without irreversible changes if the accelerating voltage is appropriately set in SEI mode. Since the images of (j) were quite different from the previous findings, the reproducibility of these images was carefully validated.

**Figure 6:**
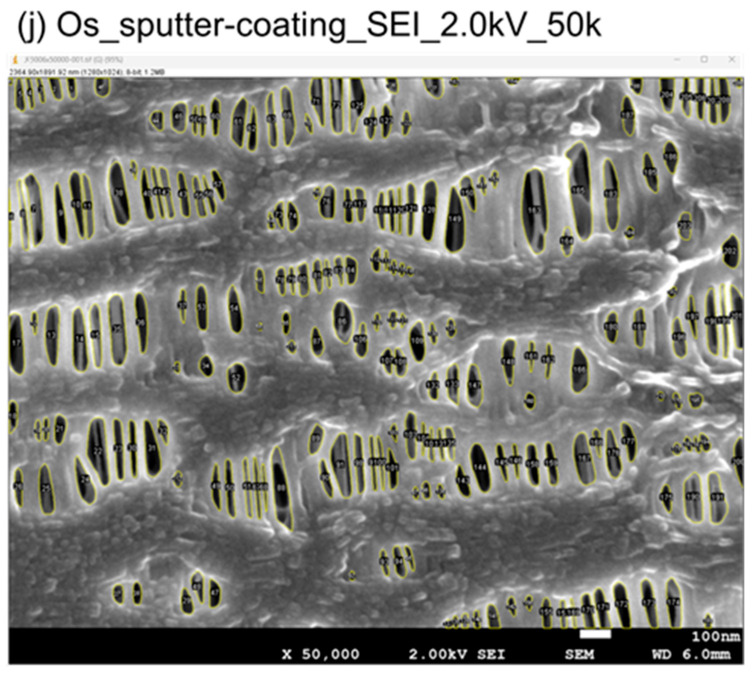
Image analysis of the sample (j).

Figure 6 shows the example of actual analyzed images of Figure 5(1) (j). The openings on the innermost surface of the membrane were visually checked and numbered one by one to determine if they were pores or not. The area and the circle equivalent diameter were calculated by trimming off the areas that could be recognized as pores one by one.

### 3.4. Outer Surface of the Membrane Coated with Silicone Layer

Figure 1(q), (r), (s) and (t) shows the images of the outer surface of the PP membrane coated with the silicone layer. (q) is an image of a nonsputter-coated sample in LEI mode, and (s) is an image of a sputter-coated sample with Os in LEI mode.

In both cases, the pores on the outer surface of the membrane were found to be covered by the silicone layer [7]. The convexity was observed in the (s) image more than in the (q) image.

(r) and (t) were observed by SEI mode, (r) was taken with the nonsputter-coating and an acceleration voltage of 5.0 kV [7], and (t) was taken with the Os sputter-coated sample at an accelerating voltage of 2.0 kV. (r) was smoother than the others. The features of (t) were also different from those of (s), this was thought to be because it represented a more deeper view of the sample.

### 3.5. Significance of This Study

It is important to precisely design the anisotropy of the pore structure in the cross-section of the capillary membrane to have highly controlled anisotropic functions [5,9]. Microscopic observation technology such as FE-SEM systems have evolved independently for each individual system [10,11], and it is necessary to utilize these techniques according to research needs. The results of this study are expected to further progress the prototyping and evaluation of such related membranes., and it is expected that next-generation gas exchange membranes and ECMOs with novel antithrombogenic materials will be put into practical use.

Although multiple analytical tools should be utilized to achieve a comprehensive structural analysis, it is noteworthy that the authors are able to reflect on the findings of the various sample preparations and modes in the previous study, understand the causes, and apply countermeasures, making this study highly practical. Even though we do not have other analytical instruments such as permporometry or environmental SEM (ESEM), the relationship between pore structure and gas permeability of several gases in hollow fiber membranes have been investigated which are the outcome of this study or by FIB-SEM [20,21] in future. The goal is to clarify the anisotropic gas permeation behavior in the anisotropic pore structure of ECMO membranes based on the behavior of Knudsen flow [22] and Poiseuille flow, and how it depends on the viscosity or molecular weight of the gas.

When using SEM to observe non-conductive samples, it is difficult to minimize morphological alterations caused by metal sputtering. Since PP has a lower melting point and PP hollow fiber membranes for ECMO are porous, the observed images of membrane morphology made from PP showed differences depending on the acceleration voltage and other conditions by using an FE-SEM. Therefore, we have investigated methods to minimize morphological alterations caused by Os sputtering or without sputtering. This is contrary to common sense and has not been seen in previous studies, and utilizing this method will advance research on a daily basis. Non-sputtered LEI imaging may be applicable to other porous polymer membranes with similar fragility.

## 4. Conclusions

In this study, we attempted to observe the original morphology of the porous PP membranes for ECMO. The unique LEI mode of the FE-SEM reveals the surface convex morphology of the porous PP membrane for ECMO. Although it is possible to obtain stable images by the Os sputter-coating on non-conductive materials, the non-sputtering method is also worthwhile for the porous and fragile structure of the PP membrane with a lower melting point. On the other hand, the morphology of the deep structure of the porous PP membrane is confirmed by utilizing the SEI mode at an appropriate accelerating voltage. It is noteworthy that the authors are able to reflect on the findings of the previous study, understand the causes, and apply countermeasures, making this study highly practical. The achievements and approaches of this study are being applied to the development of next-generation gas exchange membranes.

## Data Availability

The data that support the findings of this study are available.
